# Modeling Flood-Induced Stress in Soybeans

**DOI:** 10.3389/fpls.2020.00062

**Published:** 2020-02-12

**Authors:** Heather R. Pasley, Isaiah Huber, Michael J. Castellano, Sotirios V. Archontoulis

**Affiliations:** Department of Agronomy, Iowa State University, Ames, IA, Unites States

**Keywords:** modeling, APSIM, soybean, flooding, excess water, climate change

## Abstract

Despite the detrimental impact that excess moisture can have on soybean (*Glycine max* [L.] Merr) yields, most of today's crop models do not capture soybean's dynamic responses to waterlogged conditions. In light of this, we synthesized literature data and used the APSIM software to enhance the modeling capacity to simulate plant growth, development, and N fixation response to flooding. Literature data included greenhouse and field experiments from across the U.S. that investigated the impact of flood timing and duration on soybean. Five datasets were used for model parameterization of new functions and three datasets were used for testing. Improvements in prediction accuracy were quantified by comparing model performance before and after the implementation of new stage-dependent excess water functions for phenology, photosynthesis and N-fixation. The relative root mean square error (RRMSE) for yield predictions improved by 26% and the RRMSE predictions of biomass improved by 40%. Extensive model testing found that the improved model accurately simulates plant responses to flooding including how these responses change with flood timing and duration. When used to project soybean response to future climate scenarios, the model showed that intense rain events had a greater negative effect on yield than a 25% increase in rainfall distributed over 1 or 3 month(s). These developments advance our ability to understand, predict and, thereby, mitigate yield loss as increases in climatic volatility lead to more frequent and intense flooding events in the future.

## Introduction

It is crucial to enhance models' ability to estimate the impact of soil waterlogging on plant processes. Globally, 27% of cultivated land is impacted by flooding, resulting in over $371 billion of economic losses to crop production (Dilley et al., 2005; Zhou, 2010; Ward et al., 2013; Dold et al., 2017; Kaur et al., 2017a). With the onset of climate change, escalations in the frequency of intense rainfall events are expected to increase the prevalence of waterlogged soils and, thus, potential economic and environmental losses (Villarini and Strong, 2014; Mallakpour and Villarini, 2015; Pathak et al., 2016). Today's crop production models used for climate change assessment do not accurately account for excessive moisture (Shaw et al., 2013; Lobell and Asseng, 2017; Ebrahimi-Mollabashi et al., 2019). As a result, these models do not capture the potential impact climate change induced-flooding events or excess water may have on future yields (Li et al., 2019).

The extent of the plant response to and/or resulting yield loss from excessive moisture is dependent on the timing and duration of the flooding event as well as cultivar susceptibility. Soybeans have been generally found to be most susceptible to flood damage during early reproductive stages [around R2; (Fehr and Caviness, 1977; Scott et al., 1989; Rhine et al., 2010)]. Increases in the length of flooding escalate the intensity of these plant responses (Kaur et al., 2017a). The primary plant response to flooding is a reduction in nitrogen (N) uptake: directly caused by hypoxia reducing plant N fixation and indirectly by excess water increasing NO_3_ leaching and denitrification (Bacanamwo and Purcell, 1999a).

Simulation models are increasingly used to simulate the intricacies of complex systems, integrating the partial findings of multiple studies into a single platform to improve our understanding of the system as a whole (Shaw et al., 2013). Currently, cropping system models, however, have limited or no ability to accurately simulate a plant's response to excessive soil moisture (Shaw et al., 2013; Li et al., 2019). A model with an improved capacity to simulate waterlogging stress can be used to investigate the risk posed by flooding to crop production in climate change projections. Such an investigatory tool has the potential to significantly improve current climate change projections, as they have been found to underestimate or overlook entirely the impact of flooding on crop production (Lobell and Asseng, 2017).

The Agricultural Systems Production Systems sIMulator (APSIM) is a modular framework that allows for individual models to interact on a common interface (Holzworth et al., 2014). Like other cropping system models, however, APSIM is limited when it comes to simulating crop response to excess water. Recently, Ebrahimi-Mollabashi et al. (2019) improved the simulation of maximum root depth under excessive moisture conditions (root mean square error from 46 cm decreased to 9 cm) by adding a new function into the model. They also highlighted other areas for potential improvements (vertical root distribution, photosynthesis, phenology, leaf N concentration, N fixation, and senescence) towards developing models that better represent reality. In this paper, we address three of the identified issues: photosynthesis, phenology, and fixation.

The aim of this study was to improve the overall model's performance in waterlogged environments. More specifically, the objectives were to synthesize literature data on excessive moisture, develop new algorithms for the model, test the improved model, and quantify prediction accuracy of the improved model compared to that of the default model. A secondary objective was to apply the improved model for risk analysis related to excess moisture, wherein the question asked is whether more total precipitation or isolated extreme rain events cause larger yield loss and how this yield loss is affected across different soils and weather years. While such information is critical to understand impacts of the projected precipitation variability towards developing future resilient and profitable cropping systems, to our knowledge, such analysis is missing from the literature.

## Materials and Methods

### Model Description

#### The APSIM Model

The APSIM model is an open source field-scale cropping systems modeling platform that can simulate short and long-term soil-crop-atmospheric interactions across different environmental conditions and management structures (Holzworth et al., 2014; www.apsim.info). The software contains several crop models, together with soil water, soil temperature, and soil carbon and N models. In this project, we used the soybean crop model (Robertson et al., 2002), the SWIM model for water dynamics (Huth et al., 2012), the soiltemperature2 model, and the coupled carbon and N model (Probert et al., 1998). The APSIM model is largely used to address aspects of cropping systems around the world and continue to evolve with science (e.g. Ebrahimi-Mollabashi et al., 2019) and software improvements (Holzworth et al., 2015). The crop model simulates potential water-limited and water/N limited production situations. Water and N stress modifiers are used to decrease potential production to attainable levels. Here, we focus on the water stress functions used in the soybean model, which is part of the APSIM Plant modeling framework as that of many other crop models (Wang and Smith, 2004). In this project, we used APSIM version 7.9.

#### Description of Water Stress Functions Used in APSIM-Soybean

**Figure 1:**
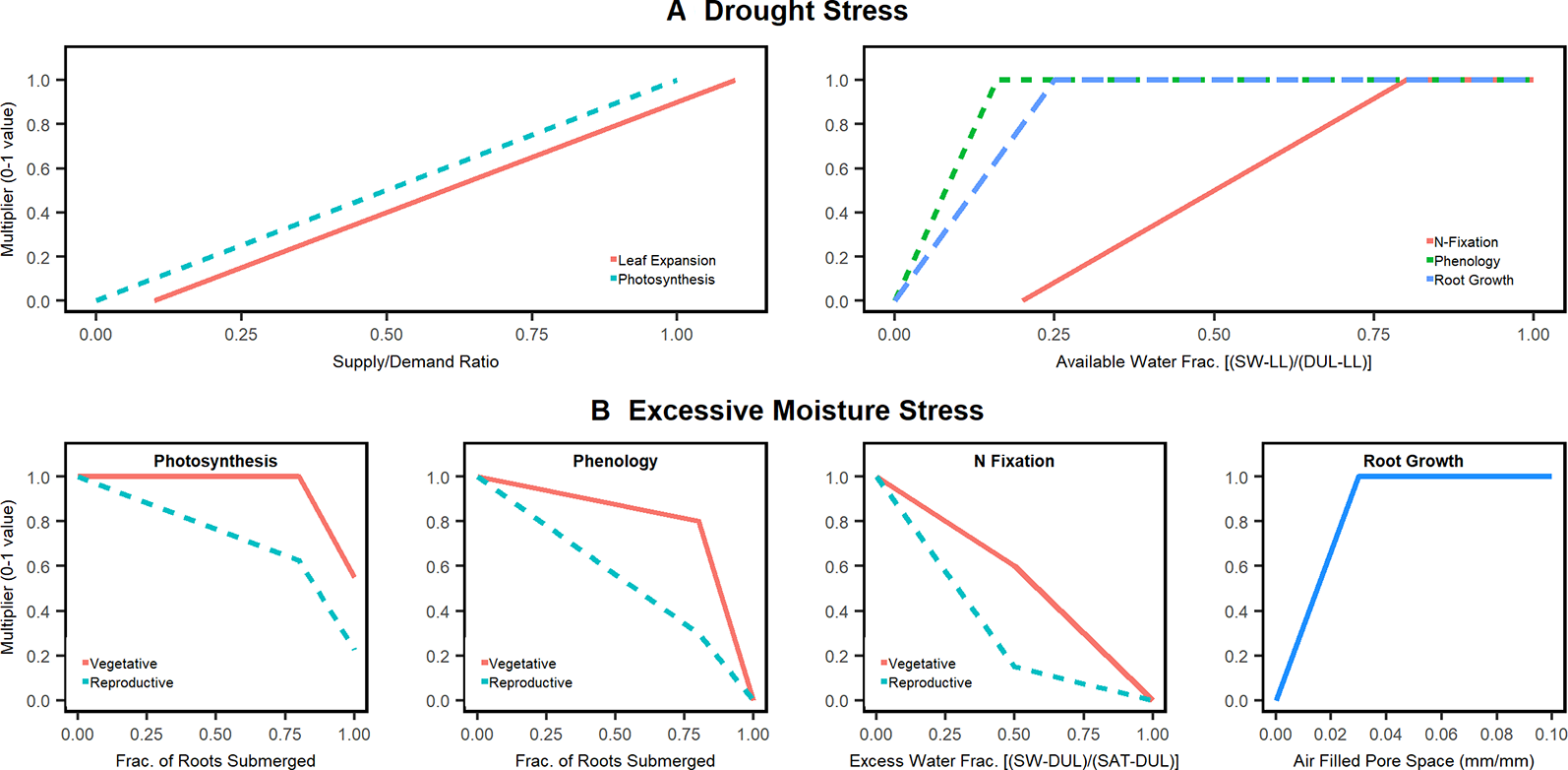
Moisture stress factors in the APSIM soybean model. **(A)** drought stress factors already present in APSIM. **(B)** Excessive moisture stress factors for photosynthesis, phenology, and fixation added and tested in this study with the exception of root growth which was already part of APSIM 7.9.

The APSIM model has a series of drought stress functions that can potentially affect photosynthesis, leaf elongation and senescence, phenology, root growth, and N fixation (**Figure 1A**). The water stress functions are based on 0–1 multipliers in the form of “x/y pairs” (Holzworth and Huth, 2009), where x is the independent variable (e.g. soil moisture) and y, the response variable (e.g. photosynthesis). The independent variable, x, is calculated in different ways: for photosynthesis and leaf expansion, a water supply/demand function is used to calculate stress; whereas for N fixation, water supply relative to soil water field capacity is used to calculate stress. Water supply is calculated by summing plant-available soil water content through the effective root-zone (Wang and Smith, 2004). Water demand is calculated by converting potential crop growth rate into water demand by using a transpiration efficiency coefficient normalized for vapor pressure deficit. Should the soil water supply to demand ratio fall below 1, there is drought stress.

In addition to drought stresses, APSIM soybean currently has excessive moisture modifiers for root growth and photosynthesis (radiation use efficiency, RUE; **Figure 1B**). Recently, Ebrahimi-Mollabashi et al., 2019 showed that a 3% air fill pore space value is sufficient to simulate the inhibitory role of excess moisture on root growth. For RUE, there is no detailed calibration, but typically users set the stress function to be activated when 80% of the roots are under water. None of the above stress modifiers account for the effect of crop stage. Further the APSIM SWIM model cease resource (water and nitrogen) uptake from soil layers saturated with water. Resource uptake is allowed only from the unsaturated soil layers.

### Enhancing Excess Moisture Representation in the Model

We sourced data from five greenhouse and field experiments spanning diverse environments across the US to develop a database to support model enhancement. The selected studies had time series data and yield response to flooding events (varying in event duration and timing, see **Table 1**), including control plots with no stress. We first set up APSIM to simulate the control plots by utilizing public data sources to create soil and weather files (see below). We then reviewed literature on excess moisture impacts to conceptualize new functions and finally, implemented and tested the new functions in APSIM using the dataset listed in **Table 1**. Model improvement quantified by comparing the default APSIM 7.9 version with the new version developed here.

**Table 1:** Overview of the published studies used in this modeling study.

Experiment/Test Number	References	Year	Location	Latitude (°N)	Soil Type	MG	Flooding Treatment	Experiment Type	Data Used for Calibration/Testing
**Calibration Database**									
1	Scott et al., 1989	1987	Keiser, AR	35.7	Sharkey Clay	4	Flooding for 2, 4, 7, and 14 days at V4 and R2	Field/Duration	Biomass, yield
2		1987	Stuttgart, AR	34.5	Crowley Silt Loam	4			
3	Scott et al., 1990	1989	Keiser, AR	35.7	Sharkey Clay	4	Flooding for 7 days at V1, V4, and R2	Field/Timing	Plant nitrogen, biomass, yield
4	Board, 2008	1999–2000	Baton Rouge, LA	30.4	Mhoon Silt Loam	5	Flooding for 7 days at V4, R1, R3, and R5	Open-ended outdoor greenhouse /Timing	Yield
5	Rhine et al., 2010	2003–2004	Hayward, MO	36.4	Sharkey Clay	4	Flooding for 8 days at V5, R2, and R5	Field/Timing	Yield
**Testing Database**									
1	Unpublished Data from Archontoulis et al., 2020	2018	Ames, IA	42.0	Upshur Silty Clay	3	Periodic waterlogged conditions	Field	Plant nitrogen, biomass, yield, LAI
2	Oosterhuis et al., 1990	1986	Stuttgart, AR	34.5	Crowley Silt Loam	5	Flooding for 4 days at V4 and R2	Field	Biomass, Yield
3						5.5			

#### New Algorithm Development

Various studies have found that excessive soil water limits water and N plant uptake and dry matter allocation to leaves (Sallam and Scott, 1987; Oosterhuis et al., 1990; Scott et al., 1990; Bacanamwo and Purcell, 1999a; Rhine et al., 2010) as well as root growth (Ebrahimi-Mollabashi et al., 2019), leaf size (Bacanamwo and Purcell, 1999b), and N fixation (Thomas and Sodek, 2005; Santachiara et al., 2019). Not all of these processes, however, are currently captured in APSIM. To enhance APSIM to simulate additional excessive moisture impacts we developed three algorithms to adjust photosynthesis, phenology, and N fixation rates when soil water exceeds field capacity (the new functions are termed oxdef_photo, oxdef_pheno, oxdef_fix, respectively, where “oxdef” stands for “oxygen deficit”; **Figure 1B**). The algorithms expand upon the limits of the drought algorithms wherein x = 0 when the soil is at field capacity (no stress) and x = 1 when the soil is saturated (full stress).

#### Excess Moisture Stress on Radiation Use Efficiency (Photosynthesis)

Flooding results in the inhibition of photosynthetic processes in the mesophyll, photoassimilate transport in the phloem, and of gas conductance and, thus, in a reduced photosynthetic rate (Oosterhuis et al., 1990; Mutava et al., 2015). Modeling the photosynthetic processes at such detailed level is beyond the scope of this study; we followed a more general approach to represent the phenomenon at a higher scale (canopy photosynthesis). On a canopy level, Oosterhuis et al. (1990) found a 16%–33% reduction in net photosynthesis after 48 h of flooding at V4 stage (4^th^ leaf) and a 22%–32% reduction at R2 stage (early reproductive stage). Mutava et al. (2015) found a 28%–39% reduction after 15 days of flooding initiated around V6 (6^th^ leaf).

The culmination of our literature review resulted in the development of the oxdef_photo function response curve similar to that illustrated in **Figure 1B**. The stress response to excess soil moisture was expected to be minor until 80% of the root systems is under water.

#### Excess Moisture Stress on Crop Phenology

The parameterization of the phenology stress function required stage-specific data regarding flood-induced delays in crop development (**Figure 1B**). The oxdef_pheno parameter response function was calculated using staging and harvesting date information sourced from experiments 1, 2, 4, and 5. In general, it follows the principles used in the oxdef_photo function.

#### Excess Moisture Stress on N Fixation

Previous studies have found that N fixation and, thus, plant N uptake are severely reduced under flooded conditions (Bacanamwo and Purcell, 1999a; Bertolde et al., 2012; Martínez-Arcántara et al., 2012). Bacanamwo and Purcell (1999a) found that N fixation is significantly more sensitive to flooding than biomass accumulation (and thus photosynthesis). Seven days of flooding reduced stem N uptake in N-fixing plants by 46%–67% when initiated 28 days after sowing (Bacanamwo et al., 1997). Córdova et al., 2019 found that N fixation in soybeans in Iowa, US accounts for 47% (ranging from 35 to 70%) of total aboveground N accumulation.

Data sourced from experiment 2 on plant N uptake during and after flooding treatments were used to calibrate the oxdef_fix parameter response function (**Figure 1B**). Bacanamwo et al. (1997) found that N fixation was reduced by 30% when the soil oxygen level was reduced by half and so, in our parameterization, we expected the effect of soil waterlogging on N fixation to be minor until x = 0.5.

Waterlogging reduces root respiration and soil N transformations (Ponnamperuma, 1984). The majority of active nodules are in the top 0.4 m of soil profile (Grubinger et al., 1982). In light of this, the oxdef_fix function is only activated when soil water conditions exceed field capacity in the top 0.45 m of the soil profile. During algorithm calibration, we confirmed that a depth of 0.45 m captured the N fixation activity well.

We also tested two different excess water stress functions for N-fixation: (1) percent root submergence similar to photosynthesis and phenology and (2) fraction of 0–45 cm moisture between field capacity and saturation. The second approach provided the best results and thus adopted. In the oxdef_photo, and oxdef_pheno parameters, the extent of soil waterlogging stress is relative to how the extent to which plant roots are submerged in water. Meanwhile, the oxdef_fix stress is calculated in response to soil water filled pore spaces in the top 0.45 m of soil. The depth, however, was programmed to be user defined to enable further research on the topic.

#### Stage Dynamics

The review of literature revealed that excess water impact plant processes differently depending on when the stress occurred. Thus, a stage factor was included in our oxdef functions. Flooding during the vegetative stages can result in a 17%–43% reduction in yield in contrast to 50%–56% reduction when flooding stress applied during the pod filling stages (R2–R4) (Oosterhuis et al., 1990). While the photosynthetic rate and N fixation decrease under flooded conditions at all stages, the plant can fully recover by pod filling stages if the stress is applied during the vegetative stages (Jung et al., 2008). Soybeans subjected to flooding during pod filling stages are unable to fully recover their preflooding photosynthetic and fixation rates following a flooding event before reaching maturity (Scott et al., 1989; Rhine et al., 2010). Moreover, N fixation peaks during pod filling period (between R3 and R5) (Zapata et al., 1987; Martínez-Arcántara et al., 2012; Córdova et al., 2019). Therefore, the photosynthesis, phenology, and N fixation algorithms needed to be more sensitive during the reproductive stages. In light of this, we applied a stage dependency on the functions such that the plant's response to waterlogging stress is more extreme during the reproductive stages (**Figure 1B**). The stage dependency component also provided the model with more flexibility with which to fit experimental observations and perform scenario analysis regarding the susceptibility of cultivars to excess water stress. The crop stage at which the plant is more sensitive to excess water is a user defined parameter.

### Model Calibration

#### Calibration Databases, Soil Data, and Weather Data

Grain yield, biomass, and plant N data from five published controlled flooding experiments (details listed in **Table 1**) were used to calibrate the new developed algorithms and quantify improvements in model accuracy. Information about the management strategies and cultivars were drawn from the published text and tables (see references in **Table 1**). Georeferenced daily weather data (minimum and maximum temperature, precipitation, and average radiation) was sourced from NASAPOWER (https://power.larc.nasa.gov/) (**Supplementary Table 1**). Soil data was sourced from the SSURGO database (Soil Survey Staff, 2019) and converted to APSIM format using the approach followed by Archontoulis et al. (2014). The lack of specific (local) soil and weather data from each experiment probably caused some error in the simulation process and reduction in prediction accuracy. The soil profiles used are provided in **Supplementary Table 2**. When more soil data were available in the studies listed in **Table 1**, we used them to further test APSIM processes, e.g., simulation of soil water dynamics, to ensure that the model reflect reality well. We simulated flooding by adding excessive amounts of rainfall until the water table reached the surface during the flooding treatment periods detailed in the studies. In-season soil water pressure measurements from experiment 3 were used to confirm APSIM's ability to accurately simulate fluctuations in soil water levels (**Supplementary Figure 1**). Details of cultivar parameters used to model each of the calibrating experiments are listed in **Supplementary Table 3**. Photosynthesis and fixation algorithms had to be customized for each of the calibration experiments with the exception of experiments 1 and 2 (**Supplementary Figure 2**). The parameters used for the five calibration experiments were then averaged to generate “default” algorithms that were then used in the model evaluation and sensitivity analysis.

### Model Evaluation

#### Testing Databases

Three independent datasets were used to test the model accuracy. The first testing dataset was comprised of unpublished grain yield and time series biomass, leaf area index (LAI), and plant N data from three plots in one of Iowa State University Forecast and Assessment of Cropping sysTemS field experiments that were flooded periodically throughout the growing season in 2018 (Archontoulis et al., 2020); the second and third datasets were comprised of grain yield and time-series biomass data from 2 published controlled flooding studies (Oosterhuis et al., 1990). Details of these studies are listed in **Table 1**.

#### Statistical Metrics

To calibrate the parameters and test the model's accuracy in capturing grain yield and above-ground biomass, we compared the simulated data with the observed data. The statistical analysis of the improved model's performance was conducted using R software (RStudio Team, 2015). Goodness of fit analysis was measured by calculating relative root mean square error (RRMSE) and modelling efficiency (ME) (see Archontoulis and Miguez, 2015 for equations). A lower RRMSE value and a higher ME value correspond to greater accuracy in the simulations. In the calibration process, these metrics were calculated for yield and biomass data using measured and simulated normalized values relative to the controls in each experiment. Normalizing the data allowed us to compare crop response to flooding across multiple environments by minimizing the effect of any covariates.

A robust model must be able to respond appropriately to variation in weather, soil, and management. In order to test the robustness of the improved APSIM soybean model, we expanded the scope and conditions of the five simulated experiments beyond their studied timescale and climate. First, we ran the experiments with their designated flooding treatments for 30 years (1988–2018), resetting the soil organic matter and N levels on January 1 of each year with the oxdef algorithms inactivated and then with them activated. We then tested the differences between the original and new model in yield and biomass results using a boxplot analysis.

### Sensitivity Analysis

To quantify the impact of each new function in the simulation process and how sensitive each parameter is, we performed a sensitivity analysis. In this analysis, we used weather, soil, and management data from Experiments 1 and 2 (**Table 1**). We ran the model for 20 years (1991-2011) with an annual reset of soil and surface organic matter and initial water/nitrogen conditions on January 1 of each year. In each simulation, we changed the parameters of one oxdef algorithm ± 1 and ± 2 standard deviations from the default values (see **Figure 1**). For oxdef_fix, the y value at x = 1 (where the soil is saturated) was not adjusted for this analysis in accordance with existing literature which has consistently found that N fixation stops (y = 0) when soils are fully saturated. The variable outputs were averaged over all flooding treatments within each experiment, but analyzed for each experiment separately because soil type played a significant role in determining the sensitivity of some variables to variation in the parameters (P < 0.05).

### Risk Analysis

To explore the potential impact of excess water in climate change research, we ran the control simulations of the 5 calibration experiments for 30 years (1988–2018 with the soil organic matter and N levels reset on January 1 of each year), altering the rainfall patterns. Climate change scenarios were generated in three different ways: (1) increasing rainfall by 25% for June, July, or August separately; (2) increasing rainfall by 25% for June, July, and August together; and (3) adding extreme daily rain events of 50 mm at varying frequencies throughout the season (one, two, three, and four times in June, July, and August with the timing evenly distributed throughout each month). In total, these eight climate scenarios were meant to quantify estimate risks associated with extreme rain events (amount and timing during crop growth) on soybean yields and also demonstrate how the improved model can be used to inform agronomists about the impact of future climate change-induced alterations to our weather pattern.

## Results

### Model Calibration

**Figure 2:**
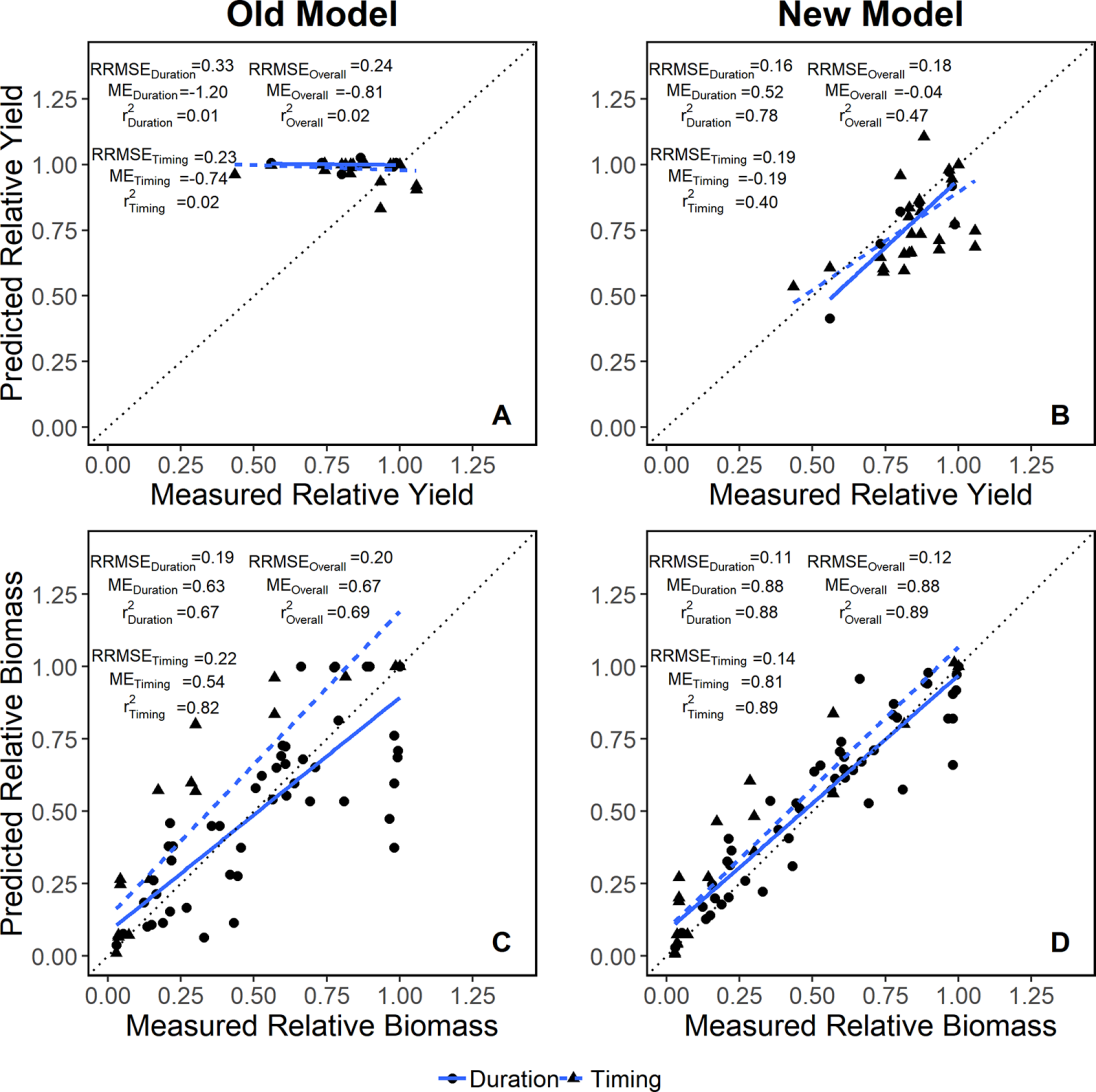
Model evaluation for yield **(A, B)** and biomass **(C, D)** data in both flooding duration (solid blue line) and timing (dashed blue line) studies relative to a 1:1 reference line (dotted black line). Relative root mean square error (RRMSE), modeling efficiency (ME) and R^2^ for each dataset are included in the panel.

The addition of the *oxdef* algorithms improved APSIM's accuracy in simulating yield and biomass response to flooding events (**Figure 2**). When the dataset was divided into those looking at flood duration (experiments 1 and 2) vs. flood timing (experiments 3, 4, and 5), RRMSE and ME improved in all cases, however, the improvements were more pronounced for the flooding duration (**Figure 2**). The model accuracy (measured as r^2^) increased by 0.45 overall, 0.77 in duration studies, and 0.38 in timing studies for yield (see **Figure 2**).

**Figure 3:**
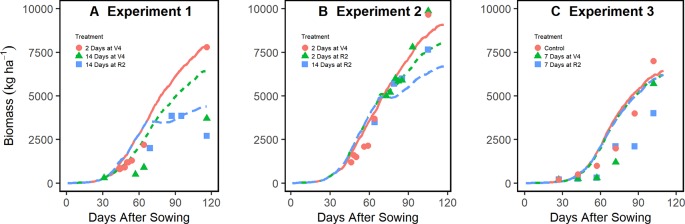
In-season simulation of biomass accumulation under different flooding treatments in experiments 1 **(A)**, 2 **(B)**, and 3 **(C)** (all located in Arkansas, experiments 1 and 3 on Sharkey Clay soil and experiment 2 on Crowley Silt Loam soil; see **Table 1** for more details). Lines represent APSIM model simulations, and points, the measured data from the experiments.

**Figure 4:**
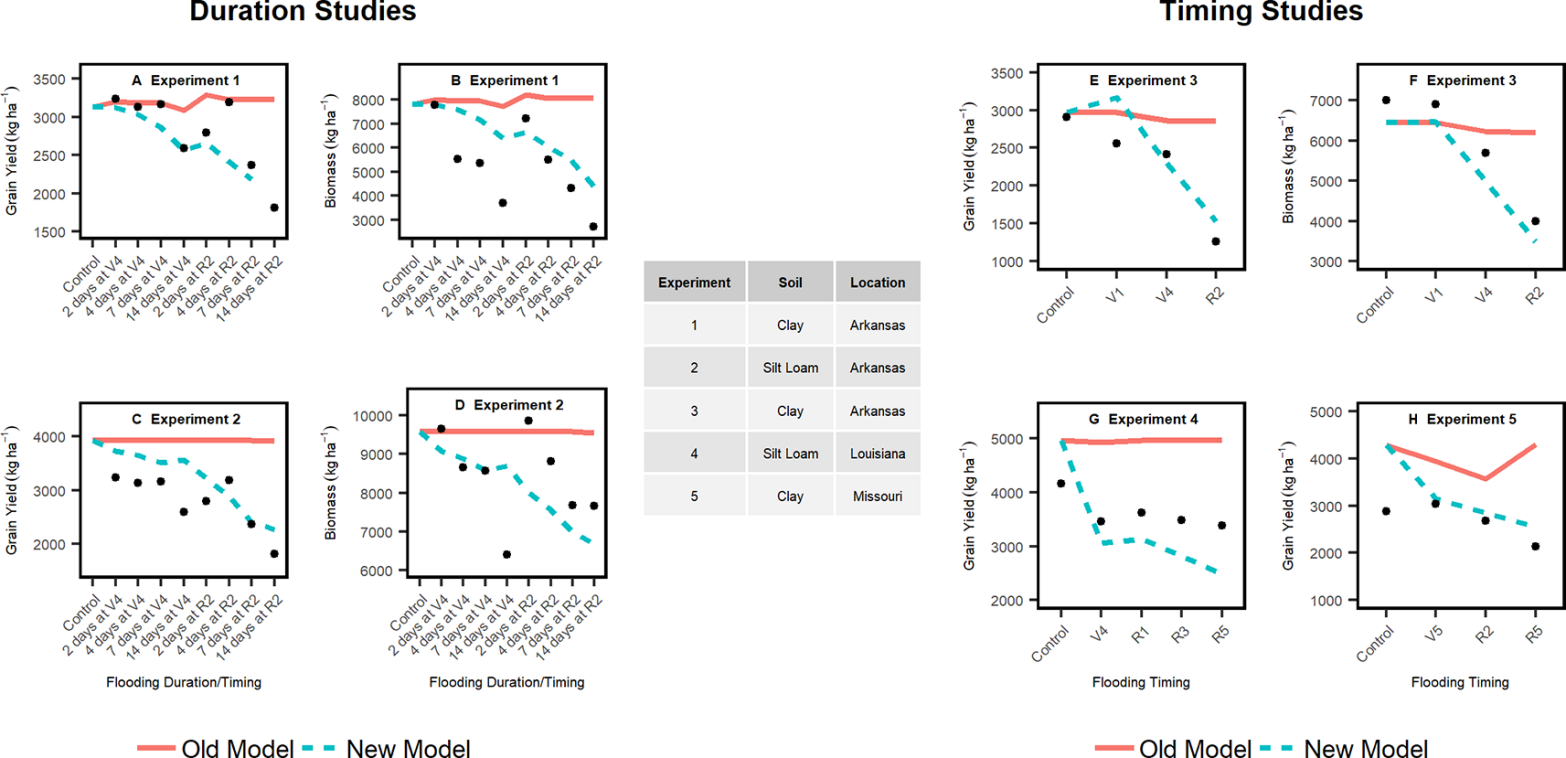
Simulated grain yield and biomass with the improved and original APSIM model (lines) versus experimental data (filled cycles) from flooding duration (left panels) and flooding timing (right panel) studies. The flooding events for the duration studies **(A–D)** were initiated at V4 (4^th^ leaf) and R2 (early reproductive stage). The flooding events for the timing studies **(E–H)** were initiated at V4 (4^th^ leaf), R1 (Beginning of reproductive phase), R3 (beginning of pod filling period), and R5 (end of pod filling period).

**Figure 3** demonstrates that the enhanced model simulated how flooding events reduce biomass accumulation during the season for experiments 1, 2, and 3. The model effectively slowed photosynthesis and stalled phenological development during flooding events as evidenced by the temporary reduction in the rate of biomass accumulation (**Figure 3**). The improved model was able to capture the at-harvest yield and biomass response to different flooding treatments better than the original model across multiple soil types and climates (**Figure 4**). The improved model also captured the flood-induced depression of N fixation and root growth found in the literature (**Supplementary Figure 3**). In general, yield results from this improved soybean model were significantly different from the original model (P < 0.001) (**Supplementary Figure 4**).

In the improved model, lengthening the flooding duration from 2 to 14 days at the V4 stage (4^th^ leaf) only decreased the yield by 2% in contrast to more than 50% at R2 (early reproductive stage). In the 30-year simulation, the improved model simulated 2% to 16% greater yield loss each year from flooding during the vegetative stages than the original model (**Supplementary Figure 4**). Flooding events during the reproductive stages reduced yield, on average, up to 61% more in the improved model relative to the default.

### Improved Model Evaluation Against Independent Dataset

**Figure 5:**
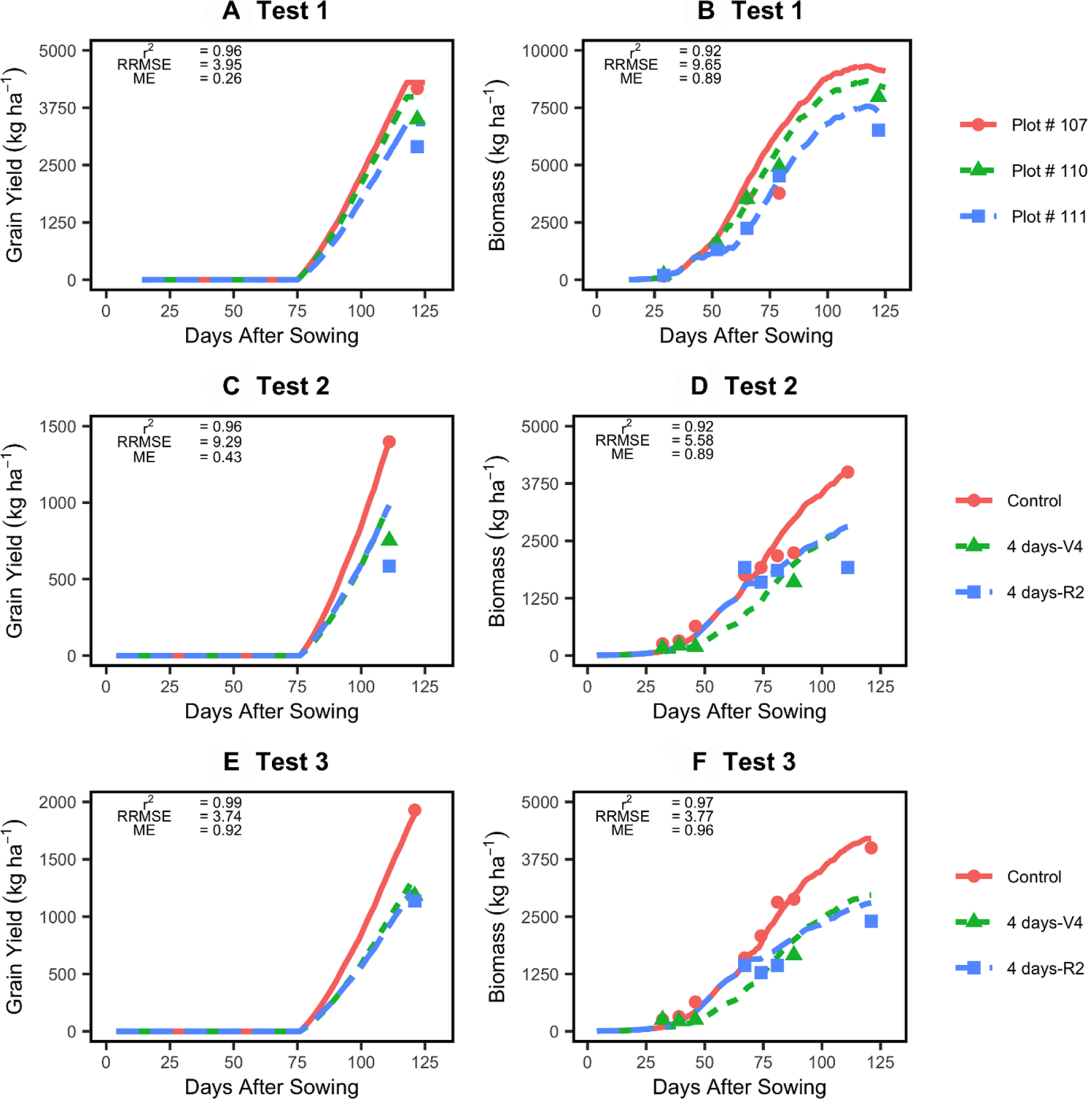
Testing of the improved model (lines) against two independent datasets (points). Left panels show model evaluation for yield **(A, C, E)** and right panels model evaluation for in-season biomass accumulation **(B, D, F)**. See **Table 1** for experimental details.

When tested on three independent datasets, the model was able to simulate yield and biomass accumulation accurately (**Figure 5**). The r^2^ values consistently exceeded 0.9, and the RRSME values were low (< 10; **Figure 5**). The model was able to capture delays in biomass accumulation during the flooding events as well as the final yield and biomass response to flooding events in different locations/at different stages. Similarly, time series plant N uptake and LAI data reported in the Test 1 dataset showed that the improved model was also able to capture those responses to flooding relatively well (**Supplementary Figure 5**).

### Sensitivity Analysis

**Figure 6:**
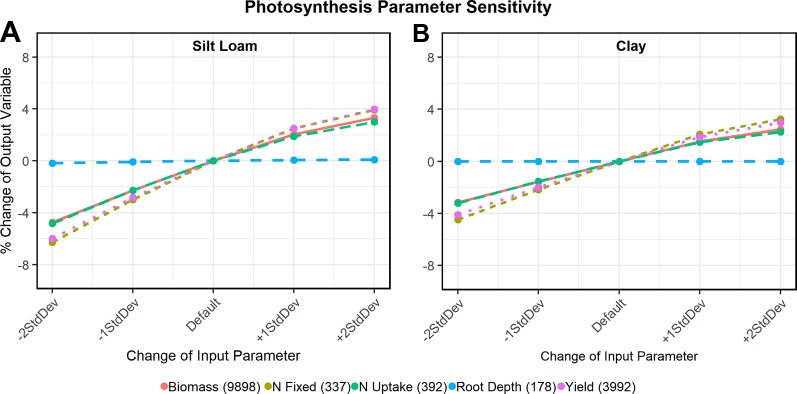
Sensitivity analysis of oxdef_photo parameters on a silt loam soil **(A)** and clay soil **(B)**. The output variables (y-axis) shown in each graph are biomass (kg ha-1), N uptake (kg ha-1), N fixation (kg ha-1), maximum root depth (cm), and end of season grain yield (kg ha-1). In the legends, the numbers in parentheses in the output variables refer to the default values.

Sensitivity analysis (**Figure 6**) of the newly developed functions showed that all tested output variables (e.g. biomass) were sensitive to changes in the oxdef_photo parameters: yield varied as much as 6% from the default value on silt loam soil and 4% on clay soil. The total amount of N fixed varied up to 6% and 4.5% on the silt loam and clay soils, respectively and total N uptake, 5% and 3%, respectively. Maximum rooting depth varied less than 1% irrespective of the soil texture. There was negligible variable response to changes in oxdef_pheno parameters and only a slight response found in the amount of N fixed and taken up by the plant (< 1%) to variation in the oxdef_fix parameters (data not shown).

### Risk Analysis

**Figure 7:**
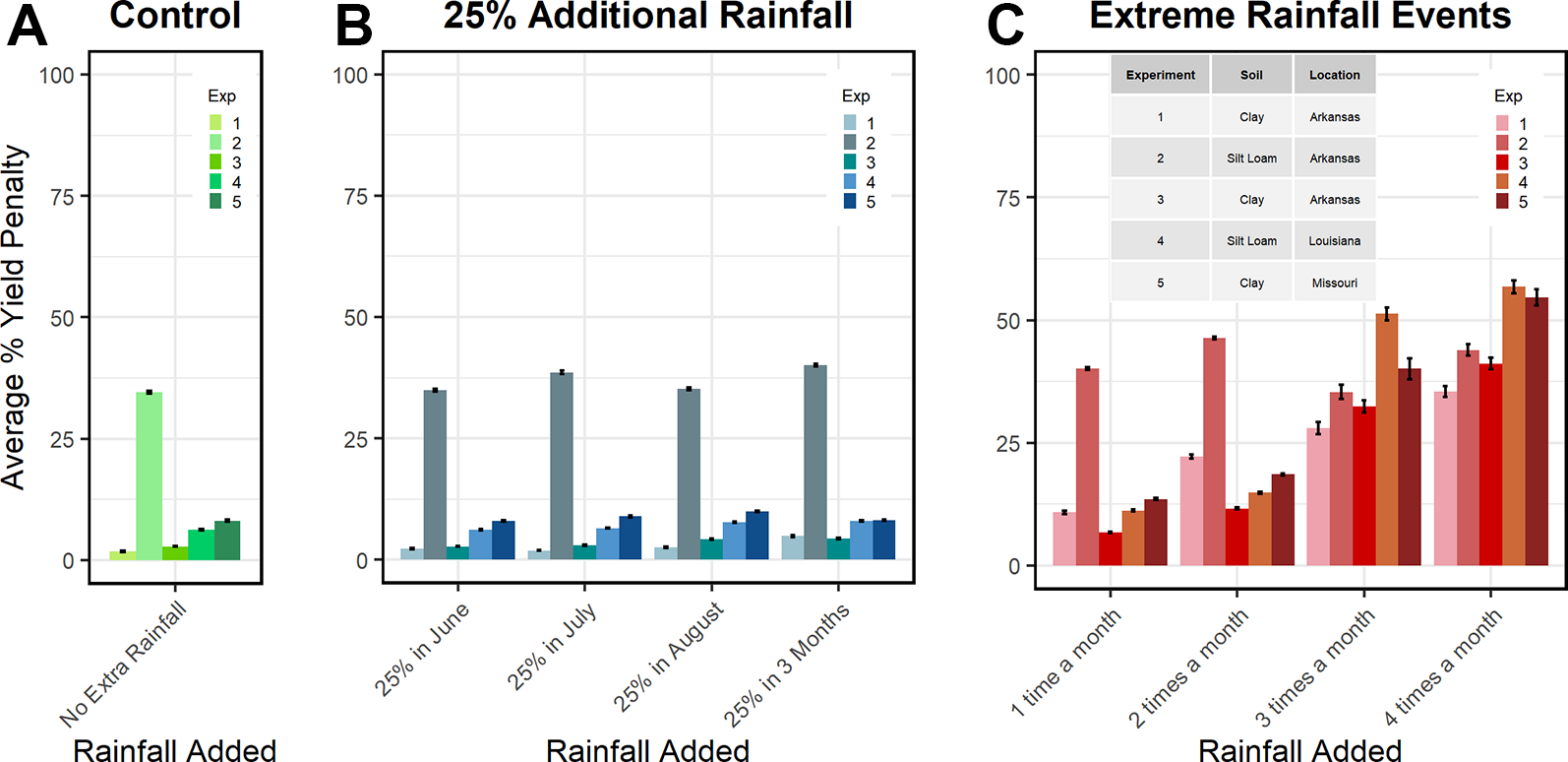
APSIM soybean simulations of soybean % yield penalty in different soils panel **(A)**, 30-year weather (1988-2018) plus 25% increased precipitation amount on soybean yields panel **(B)**, and 30-year weather plus extreme rain evens on soybean yields panel **(C)**. Soil texture for each experiment is provided in panel **C**, further details are provided in **Table 1**.

In the eight climate change scenarios, the improved model demonstrated that intense rainfall events (one to four times a month) had a greater effect on yield than a 25% increase in rainfall during any or all month(s) (**Figure 7**). On average, relative to the control, when 25% extra rainfall was applied during June, there was no negative impact on grain yield. In July and August, 25% extra rainfall depressed yields by around 1%. When 25% extra rainfall was applied for 3 months, yields decreased about 2%. Intense rain events applied 1, 2, 3, and 4 times a month resulted in yield penalties averaging 6%, 12%, 27%, and 36%, respectively. These penalties more than tripled in experiments 3, 4, and 5 when event frequency increased from 2 to 3 times a month (9%–10% to 30%–45%).

## Discussion

### Simulating Flooding Stress

We focused on modeling the impact excess water stress has on yield and biomass because the existing literature has demonstrated that crop models fail to simulate the decline in yields under excessive precipitation (Li et al., 2019). Crop models being able to simulate the full range of water stress (from too little water to too much water) is important for accurately predicting and explaining how climate change will impact crop production as climate change is expected to increase the intensity and frequency of extreme rainfall events and thus, the prevalence of flooding.

In this study, we developed and tested new algorithms within the APSIM software for excess water stress impacts on phenology, photosynthesis, and N-fixation. While the model was able to simulate root depth response to water table fluctuations (Ebrahimi-Mollabashi et al., 2019), prior to our additions, it was unable to simulate above-ground biomass, crop staging, and N fixation responses to soil waterlogging. The additions enhanced APSIM's capacity to accurately simulate waterlogging stress across a wide range of environments as illustrated in **Figures 2-4**. The impact of these improvements span far beyond the soybean model as they can be easily implemented to all crop models included in the APSIM Plant framework (about 80% of crop models).

Furthermore, the simplicity of these new stress functions makes them easily adoptable for other modeling groups or other crop models within APSIM after appropriately calibration.

The integration of detailed soil profile data in APSIM captured the significant role of soil texture in dictating stress levels in experiments 1 and 2 (**Figures 2–4**; Schipanski et al., 2010). The total N fixed and yield response variables were more sensitive to changes in the oxdef_photo function with a silt loam than with a clay soil while the total N fixed variable was more sensitive to changes in the oxdef_fix function on a clay than a silt loam soil. Sensitivity in total N fixed does not necessarily correspond to sensitivity in plant N uptake: in APSIM, the plant takes up N from the soil prior to fixing N. While the oxdef_fix function does not directly impact soil N mineralization or NO_3_ levels, it does impact these levels indirectly through reducing biomass growth and thereby increasing soil water. In general, the N mineralization process is less sensitive to excess moisture than nitrification or N-fixation (Linn and Doran, 1984). So if there is enough soil nitrogen, the N uptake will be impacted less than N-fixation. This model behavior agrees with literature studies showing that application of rescue N fertilizers mitigates flooding stress (Kaur et al., 2017b).

The models SWAGMAN Destiny (Meyer et al., 1996) and DRAINMOD (Skaggs et al., 2012) have been found to simulate waterlogging stress with the same level of accuracy as APSIM did prior to our additions, but fall short of incorporating the stage and nutrient dynamics of waterlogging-induced stress (Shaw et al., 2013; Shaw and Meyer, 2015). Currently, SWAGMAN Destiny (Meyer et al., 1996) uses the gas-filled soil pore volume to define the degree to which the soil is waterlogged (on a scale of 0-1 aeration), wherein dry matter accumulation is reduced after soil aeration remains at zero for 3 consecutive days. In this model, dry matter accumulation influences potential yield, root growth, and senescence (Shaw et al., 2013). Meanwhile, DRAINMOD (Skaggs et al., 2012), simulates excessive soil water stress using the stress day index when the water table is above 0.3 m. In DRAINMOD, waterlogging stress can delay planting date and, with the guidance of the user, limit root depth. As such, while these other models can capture some aspects of crop response to waterlogging stress, their approaches oversimplify the dynamics driving flood-induced yield penalties and thus limit their usefulness in risk scenario analysis. Should these models adopt the additions proposed in this paper, we would expect their performance accuracy to improve just as that of APSIM did.

### Implications for Climate Change

Cropping system models have long been seen as valuable tools for anticipating the impact of climate change on crop production, but the vast majority of this application has focused on how heat, elevated levels of CO_2_, and drought will impact agriculture on a global scale (Asseng et al., 2013; Ewert et al., 2015). Asseng et al. (2018) found, however, that changes in temperature and rainfall will have a much greater impact on yield than changes in CO_2_ levels. Jia et al. (Forthcoming) cites that increased temperatures under climate change will not necessarily lead to more overall rainfall, but rather to more frequent and extreme excess rainfall events. However, when models have investigated at the impact of projected increases in excess rainfall, they have primarily investigated the effect of increases in mean or total precipitation, not that of increases in the intensity of rainfall events (Ewert et al., 2015; Ray et al., 2015; Lobell and Asseng, 2017). Under this approach, these models found what we found in our risk analysis: well-distributed excess rainfall has a small impact on yield (Ray et al., 2015). Lobell and Asseng (2017) found that models that only look at seasonal changes in rainfall tend to overestimate the benefits of excessive rainfall and, thereby, underestimate the potential waterlogging-induced yield penalties. Also, there is a lack of agreement among models on how extreme rain events will impact yields at the regional scale (Orlowsky and Seneviratne, 2012). Models operating on a global scale and cannot achieve the resolution needed for a consensus to be reached as yield response to flooding is dependent on factors that vary significantly across a small landscape (e.g. soil type and initial moisture) (Kang et al., 2006; Tebaldi et al., 2006; Kharin et al., 2007; Orlowsky and Seneviratne, 2012; Rosenzweig et al., 2014; Ray et al., 2015; Lobell and Asseng, 2017). Moreover, Rosenzweig et al., 2014 noted that corn and soybean yield responses to excessive water are more sensitive to variability in soil conditions than other crops and so, need to be examine on a field rather than global scales.

As a field-scale model, our simulated scenarios found that an increase in the frequency of extreme rain events from two to three events in a month triples the yield penalty irrespective of soil conditions. Field-scaled models can also provide opportunities for agronomists and plant breeders to test how different management strategies and/or cultivars can mitigate flood-induced yield losses.

Our risk analysis also found that concurrent environmental conditions (soil type, initial soil water content, etc.) did play a role if how much extreme rainfall events depressed yields (**Figure 6**). We did not use a forecasted weather data to drive our risk analysis as the selection of climate model would bias our results (Corbeels et al., 2018): there are many climate models to choose from, each influencing the crop model in different ways, leading to high levels of uncertainty. Here, we set up simple climate change scenarios in order to look at the potential of the model to capture yield responses to extreme rain events and the legacy effects of these events happening at higher frequencies to demonstrate the improved model's usefulness to climate change scientists.

### Limitations to the Model

Some limitations of the improved model arose from the fact our model parametrization was based on profiles and weather data from public not local sources. This probably has caused some loss in prediction accuracy and, perhaps, over/underestimation of the parameters developed for the stress functions. However, this is of less concern, as our main objective was to enhance the model by adding new generic mechanisms that can be further refined in future model applications.

Our aim in developing the algorithms was to model the phenomena, not the mechanisms. We took this approach because we did not have access to detailed experimental data and wanted to keep the improvements as simple as possible. Moreover, excessive rainfall and, therefore, flooding events often coincide with hail and/or excessive wind and the resulting wet environment often coincides with plant disease. Modeling excess water is complicated; the default parameter values at times need to be slightly adjusted to better fit different environments, as we found with our calibration dataset (**Supplementary Figure 2**). Still, when we tested the default algorithms (**Figure 1**) on three independent datasets, we found the fit to still be strong (**Figure 5**). Still, this potential need for customization demonstrates the limitations of these algorithms in capturing all of the plant's dynamic responses to soil waterlogging-induced stress.

### Recommendations for Future Work

An area that requires future improvement is the simulation of N partitioning/redistribution within the plant. Parameterization of the N dynamics within the plant would require detailed data on changes in the aboveground tissue and leaf N during a flooding event. While experiments 4 and 5 provided some tissue and/or leaf N concentration data following 7–8 days of flooding at different development stages, it was not enough to understand and model N remobilization under excess moisture. More detailed data is needed on how plant N is redistributed during and immediately following flooding events varying in timing and duration.

## Conclusion

Our additions to the APSIM soybean model improved the model and have laid the groundwork for other models to follow. We captured a significant portion of the plant's dynamic response to waterlogging and demonstrated how the model can improve climate change projections. Future work should focus on mapping out more of the N dynamic responses to flooding.

## Data Availability Statement

All datasets generated for this study are included in the article/**Supplementary Material**.

## Author Contributions

HP conducted the literature review, developed the APSIM simulations, did the statistical and risk analyses, and wrote the manuscript. IH coded the oxdef functions into the APSIM source code. MC provided scientific guidance and reviewed drafts of the manuscript. SA designed the project, provided scientific and modeling guidance, and reviewed drafts of the manuscript.

## Conflict of Interest

The authors declare that the research was conducted in the absence of any commercial or financial relationships that could be construed as a potential conflict of interest.
