# Supplementary material for: Modeling Flood-Induced Stress in Soybeans
Source: Front Plant Sci. 2020 Feb 12;11:62. doi: 10.3389/fpls.2020.00062 (PMC7028700; doi:10.3389/fpls.2020.00062)
Supplement: Supplementary file 1 [file Table_1.docx]

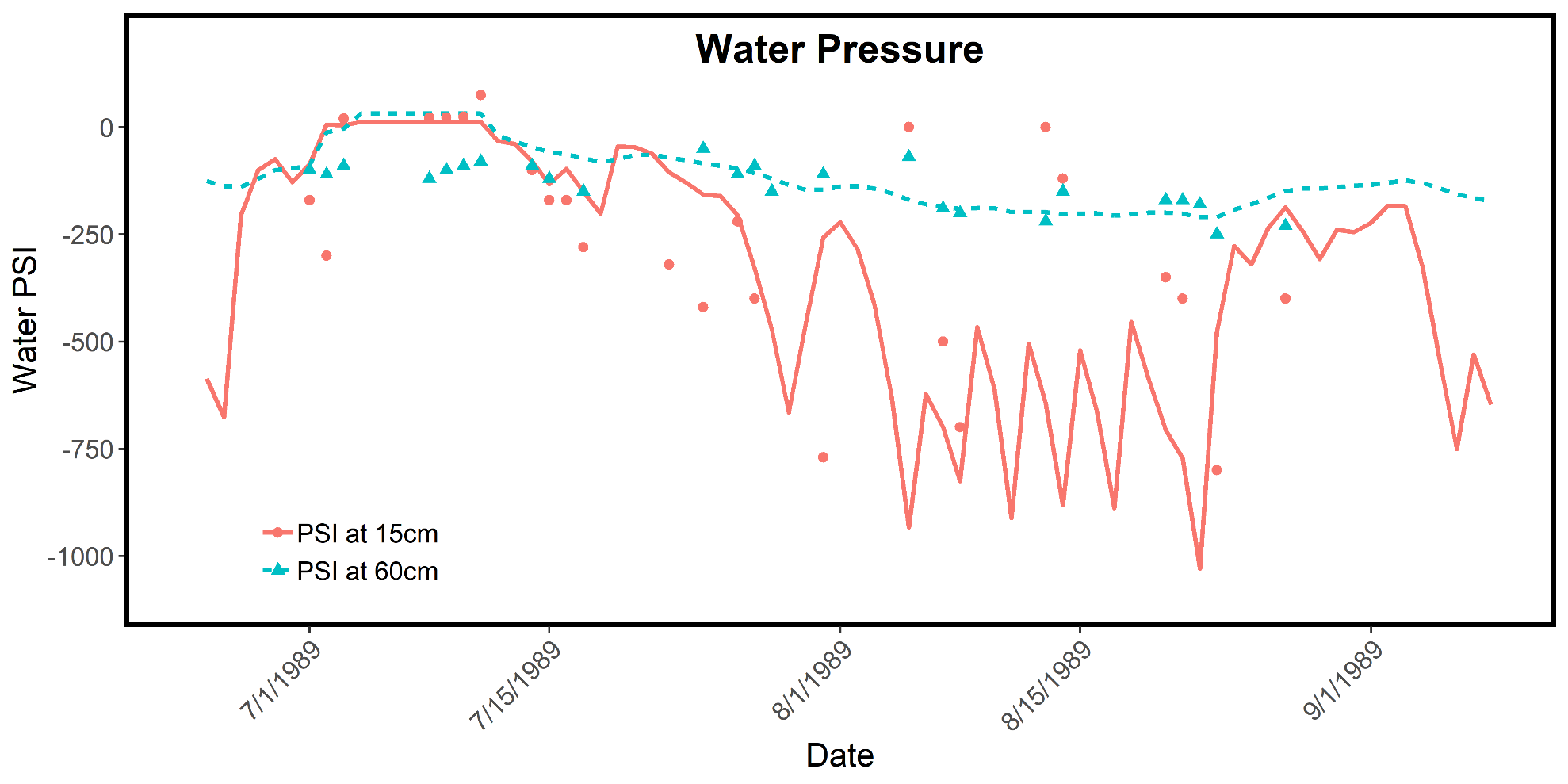


Supplementary Figure 1 Simulated (line) and actual (point) water table and water pressure (at depths 15 and 60 cm) data sourced from Nelson et al. (2011) and Scott et al. (1990), respectively.


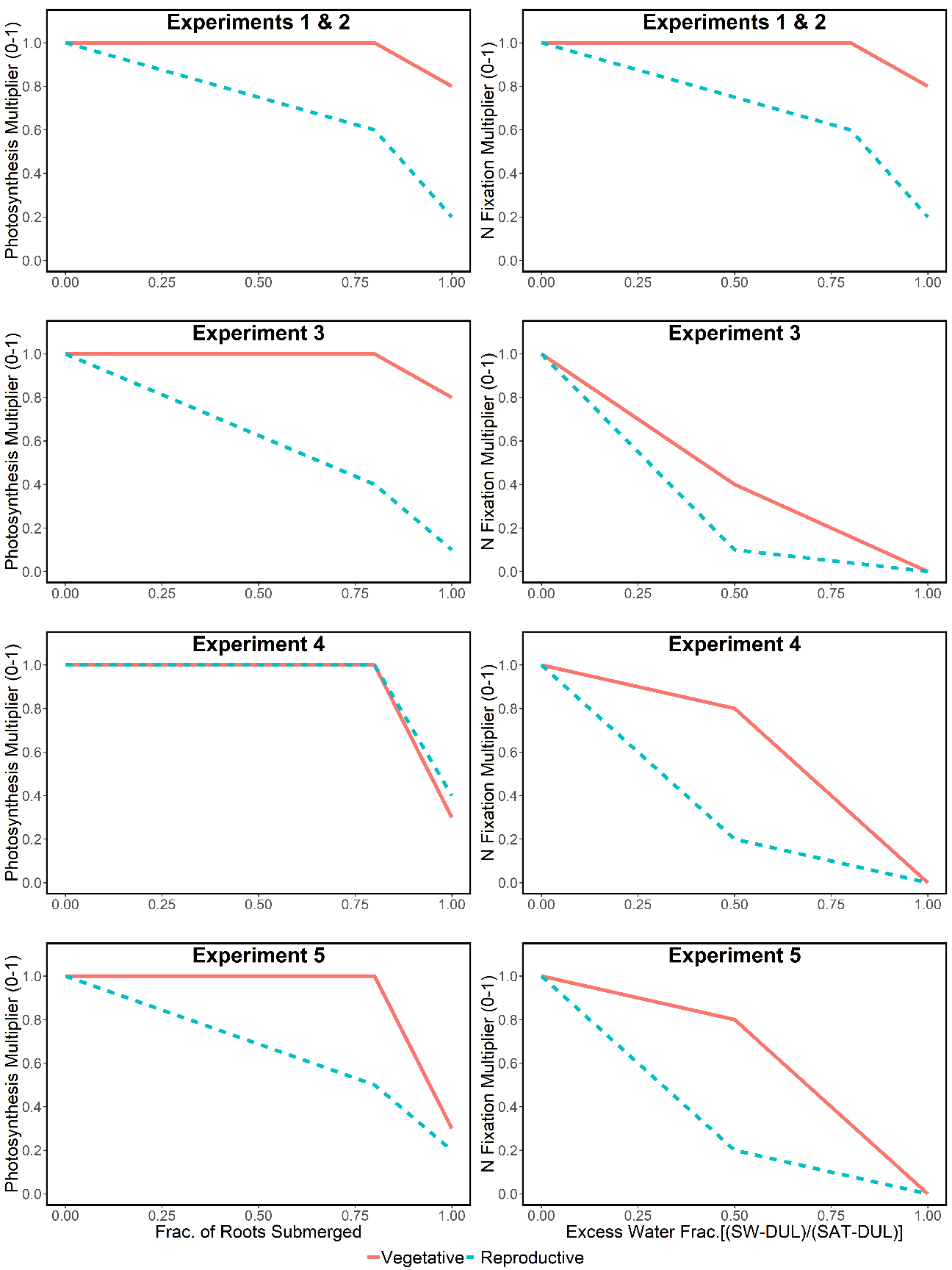


Supplementary Figure 2 Oxdef algorithms for photosynthesis and N fixation (left to right) for vegetative (solid line) and reproductive stages (dotted line) calibrated for each experiment. The photosynthesis and N fixation multipliers (y axes) are used by the multiplicative mode at different soil water stati (x axes). The phenology parameters did not have to be calibrated for each experiment, so we refer to fix XXXX (main document) for the exact values.


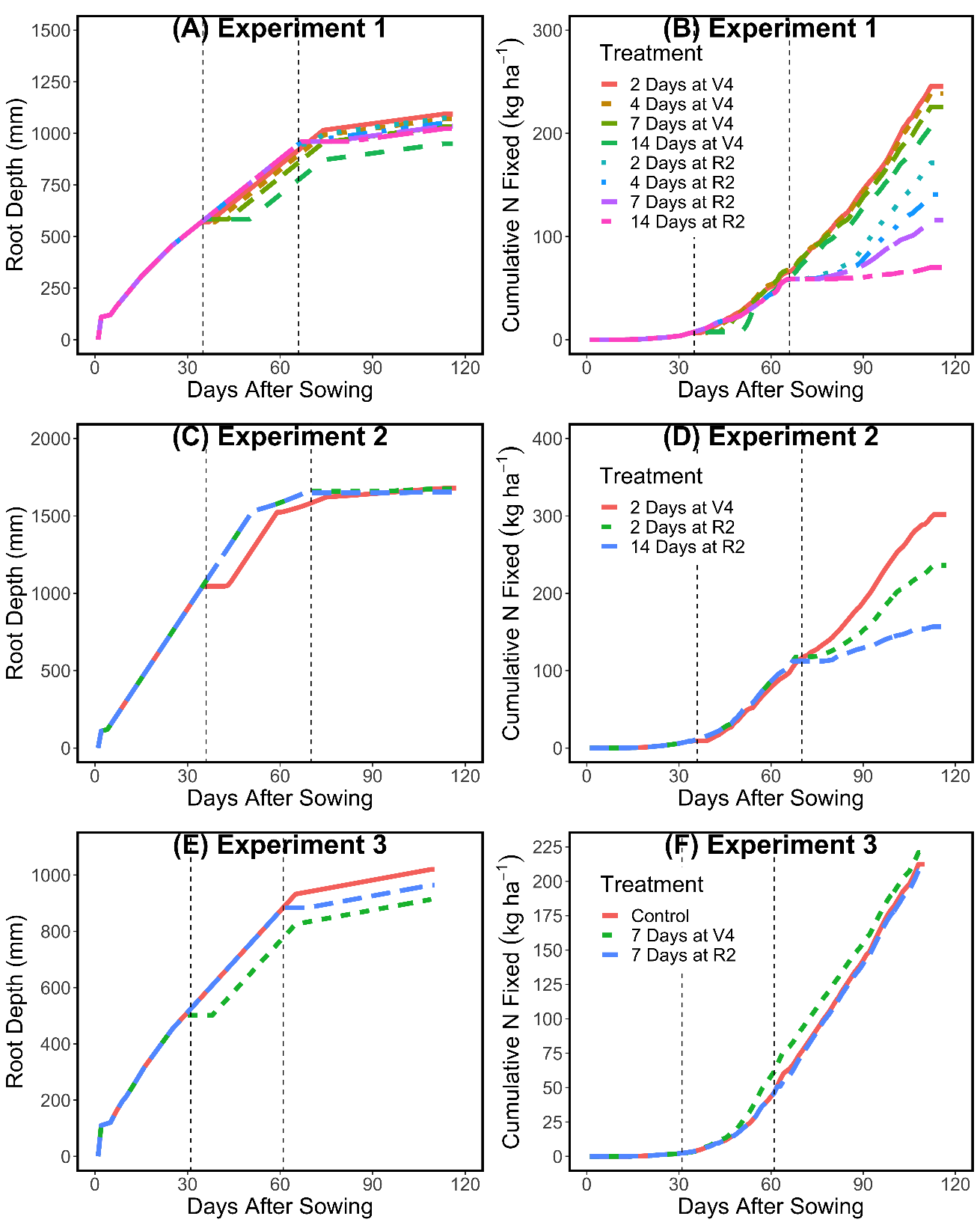


Supplementary Figure 3 Simulated root depth and cumulative N fixed in experiments 1, 2, and 3 using the improved APSIM model. Vertical dotted lines mark when flooding treatments were applied at V4 or R2 in each experiment.


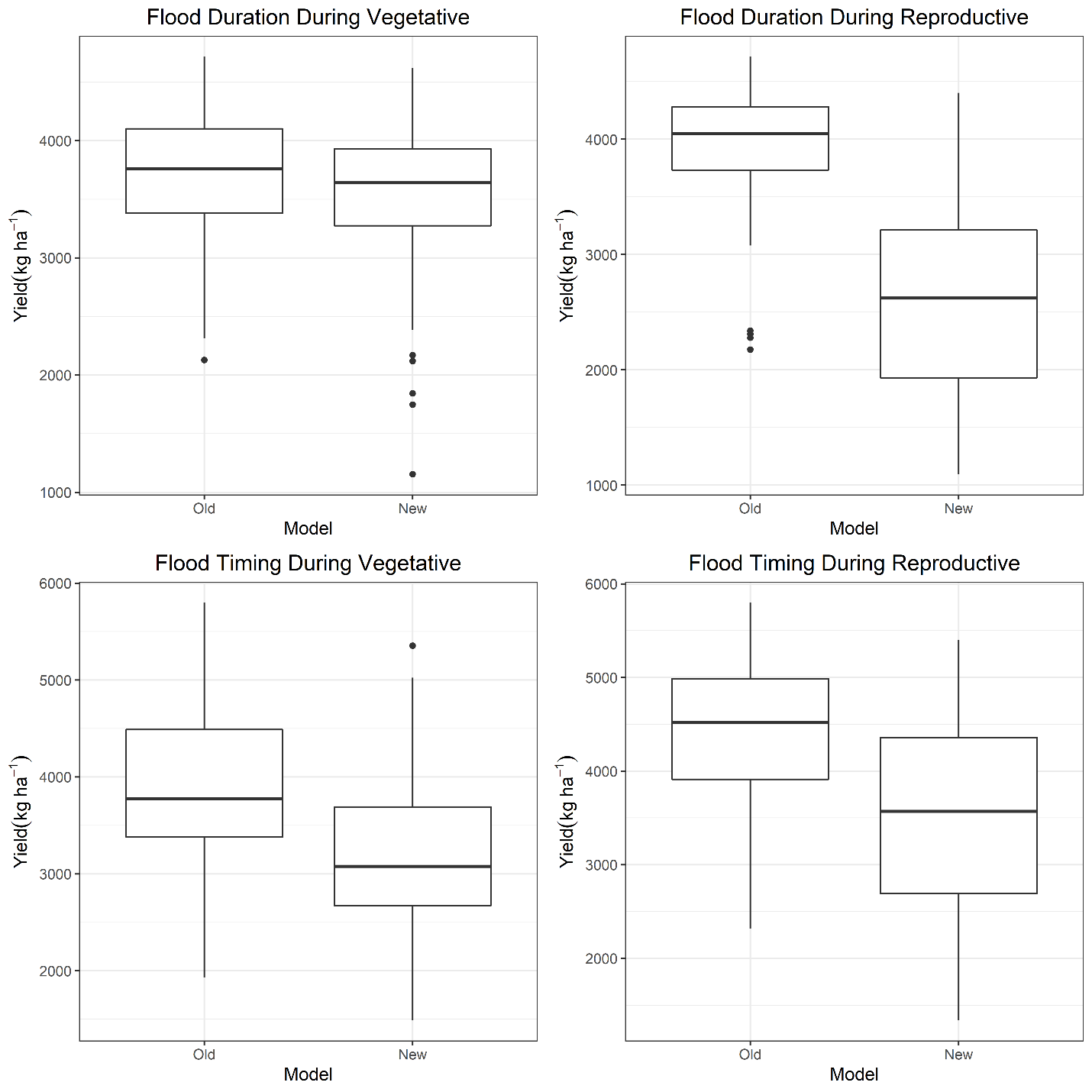


Supplementary Figure 4 Boxplot analysis illustrating how the improved model (new) behave over 30 years against the original model (old) in terms of yield prediction. The 5 calibration experiments were run from 1988-2018 with the relevant weather data for each experiment. Soil organic matter and N levels reset on January 1 of each year.


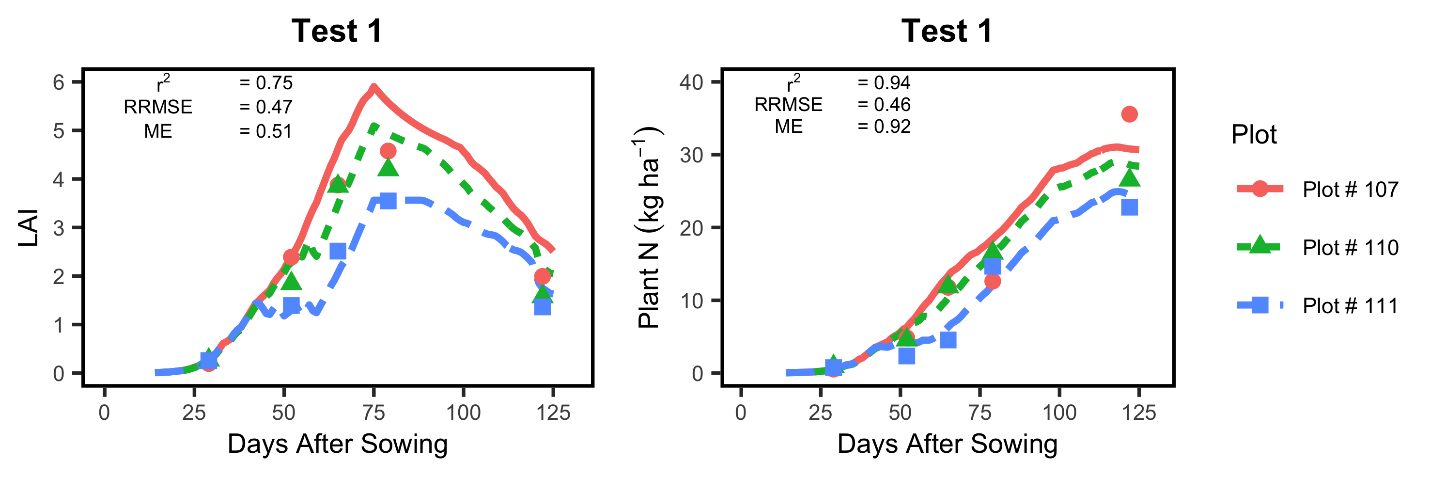


Supplementary Figure 5 Model evaluation for in-season leaf area index (LAI) and total plant N uptake in the 3 field plots that were periodically flooded throughout the growing season from one of Iowa State University Forecast and Assessment of Cropping sysTemS field experiments in Ames, IA. Lines represent APSIM simulation data and points, the measured data from the experiments.

Supplementary Table 1: Weather information by experiment, year, and month used in the simulation. See also Table 1 (main document)

|  | Maximum Temperature (°C) | | | | | Minimum Temperature (°C) | | | | | Precipitation (mm) | | | | | Radiation Sum (MJ/m^2^) | | | | |
| --- | --- | --- | --- | --- | --- | --- | --- | --- | --- | --- | --- | --- | --- | --- | --- | --- | --- | --- | --- | --- |
| Year | May | Jun | Jul | Aug | Sep | May | Jun | Jul | Aug | Sep | May | Jun | Jul | Aug | Sep | May | Jun | Jul | Aug | Sep |
| **Calibration Experiment 1 (Scott et al., 1989)** | | | | | | | | | | | | | | | | | | | | |
| 1987 | 27.3 | 30 | 32.1 | 34.7 | 31.1 | 18 | 20.1 | 21.6 | 22.5 | 17.5 | 112.1 | 102.9 | 94.5 | 57.4 | 61 | 618.6 | 685 | 655.9 | 608.6 | 542.2 |
| **Calibration Experiment 2 (Scott et al., 1989)** | | | | | | | | | | | | | | | | | | | | |
| 1987 | 27.6 | 30.4 | 33.2 | 36.5 | 31.7 | 18.7 | 20.4 | 22.4 | 23.7 | 18.1 | 135.9 | 99.8 | 68.3 | 58.1 | 83.2 | 650.5 | 677.2 | 652 | 615.4 | 534.4 |
| **Calibration Experiment 3 (Scott et al., 1990)** | | | | | | | | | | | | | | | | | | | | |
| 1989 | 24.4 | 27.4 | 29.3 | 30.8 | 26.9 | 13.3 | 19 | 21.2 | 20.6 | 15.9 | 104.6 | 173.9 | 112.2 | 45.1 | 112.9 | 590.4 | 561.2 | 612.2 | 638.7 | 468.5 |
| **Calibration Experiment 4 (Board et al., 2008)** | | | | | | | | | | | | | | | | | | | | |
| 1999 | 31.7 | 32.1 | 31.3 | 35.4 | 31.8 | 19.6 | 22.9 | 23.5 | 24.4 | 19.5 | 120.4 | 180.3 | 142.3 | 65.4 | 98 | 676.4 | 623.5 | 650.3 | 661.5 | 550.8 |
| 2000 | 33.1 | 33.8 | 34.7 | 35.2 | 31.9 | 20.7 | 23.3 | 23.7 | 24.3 | 21 | 38 | 143 | 103.5 | 83.1 | 122.7 | 706.2 | 606.9 | 707 | 679.7 | 517 |
| **Calibration Experiment 5 (Rhine et al., 2010)** | | | | | | | | | | | | | | | | | | | | |
| 2002 | 24.1 | 29.4 | 30.7 | 31.1 | 29.3 | 13.1 | 19.1 | 22.1 | 21.2 | 17.6 | 164.6 | 75.4 | 67.1 | 91.5 | 145.4 | 575.3 | 687.1 | 650 | 608.6 | 502.1 |
| 2003 | 25 | 26.8 | 30.3 | 30.7 | 25.6 | 15 | 16.7 | 20.9 | 21.2 | 14.5 | 225 | 104.6 | 117.6 | 91.4 | 106.7 | 532.9 | 626.9 | 696.1 | 604.2 | 529.5 |
| 2004 | 26.4 | 28.1 | 29.4 | 29 | 29.6 | 16.5 | 19.1 | 20.6 | 18.5 | 16.6 | 135.9 | 89.4 | 113.8 | 102.4 | 5.1 | 564.2 | 664.4 | 636.7 | 589.1 | 571.6 |
| **Testing Experiment 1 (Archontoulis et al., 2019)** | | | | | | | | | | | | | | | | | | | | |
| 2018 | 38.1 | 35.0 | 34.2 | 31.8 | 31.9 | 7.6 | 11.3 | 12.6 | 11.8 | 8.0 | 75.7 | 297.5 | 59.9 | 193.5 | 133.9 | 619.9 | 608.4 | 747.9 | 552.3 | 383.3 |
| **Testing Experiment 2 & 3 (Oosterhuis et al., 1990)** | | | | | | | | | | | | | | | | | | | | |
| 1986 | 33.9 | 36.9 | 45.9 | 39.5 | 36.4 | 8.5 | 16.4 | 20.5 | 11.3 | 14.4 | 89.5 | 136.8 | 26.2 | 66.0 | 45.2 | 588.5 | 582.3 | 752.1 | 623.5 | 491.1 |

Supplementary Table 2: Soil profile information for each experiment used in the simulation. LL=Drained lower limit, DUL=Drained upper limit, SAT=Saturation, Ksat=Hydraulic conductivity, SOC=Soil organic carbon, Fbiom and Finert=SOC fraction to BIOM and INERT pool. See also Table 1 (main document)

| Depth | LL15 | | DUL | | SAT | Ksat | SOC | Fbiom | Finert |
| --- | --- | --- | --- | --- | --- | --- | --- | --- | --- |
| cm | -----------mm/mm------------- | | | | | mm/d | % | 0-1 | 0-1 |
|  | **Calibration Experiment 1 (Scott et al., 1989)** | | | | | | | | |
| 0-30 | 0.307 | 0.434 | | 0.515 | | 158.333 | 1.170 | 0.093 | 0.466 |
| 30-60 | 0.377 | 0.483 | | 0.567 | | 31.300 | 0.600 | 0.035 | 0.814 |
| 60-90 | 0.419 | 0.510 | | 0.609 | | 49.733 | 0.163 | 0.024 | 0.862 |
| 90-120 | 0.396 | 0.495 | | 0.594 | | 52.133 | 0.143 | 0.020 | 0.902 |
| 120-150 | 0.364 | 0.476 | | 0.575 | | 52.100 | 0.080 | 0.016 | 0.949 |
| 150-180 | 0.348 | 0.467 | | 0.564 | | 50.800 | 0.020 | 0.014 | 0.999 |
| 180-210 | 0.348 | 0.467 | | 0.564 | | 50.733 | 0.013 | 0.013 | 0.999 |
| 210-250 | 0.348 | 0.467 | | 0.564 | | 50.600 | 0.000 | 0.012 | 0.999 |
|  | **Calibration Experiment 2 (Scott et al., 1989)** | | | | | | | | |
| 0-30 | 0.115 | 0.297 | | 0.434 | | 203.000 | 0.875 | 0.060 | 0.406 |
| 30-60 | 0.126 | 0.340 | | 0.456 | | 104.000 | 0.455 | 0.025 | 0.763 |
| 60-90 | 0.203 | 0.377 | | 0.494 | | 88.667 | 0.440 | 0.020 | 0.849 |
| 90-120 | 0.257 | 0.388 | | 0.478 | | 58.000 | 0.440 | 0.020 | 0.852 |
| 120-150 | 0.255 | 0.385 | | 0.473 | | 58.000 | 0.270 | 0.020 | 0.885 |
| 150-180 | 0.250 | 0.384 | | 0.472 | | 50.000 | 0.090 | 0.020 | 0.999 |
| 180-210 | 0.250 | 0.383 | | 0.469 | | 49.000 | 0.033 | 0.020 | 0.999 |
| 210-250 | 0.250 | 0.381 | | 0.466 | | 47.000 | 0.014 | 0.020 | 0.999 |
|  | **Calibration Experiment 3 (Scott et al., 1990)** | | | | | | | | |
| 0-30 | 0.307 | 0.434 | | 0.515 | | 158.333 | 1.170 | 0.093 | 0.466 |
| 30-60 | 0.377 | 0.483 | | 0.567 | | 31.300 | 0.600 | 0.035 | 0.814 |
| 60-90 | 0.419 | 0.510 | | 0.609 | | 49.733 | 0.163 | 0.024 | 0.862 |
| 90-120 | 0.396 | 0.495 | | 0.594 | | 52.133 | 0.143 | 0.020 | 0.902 |
| 120-150 | 0.364 | 0.476 | | 0.575 | | 52.100 | 0.080 | 0.016 | 0.949 |
| 150-180 | 0.348 | 0.467 | | 0.564 | | 50.800 | 0.020 | 0.014 | 0.999 |
| 180-210 | 0.348 | 0.467 | | 0.564 | | 50.733 | 0.013 | 0.013 | 0.999 |
| 210-250 | 0.348 | 0.467 | | 0.564 | | 50.600 | 0.000 | 0.012 | 0.999 |
|  | **Calibration Experiment 4 (Board et al., 2008)** | | | | | | | | |
| 0-30 | 0.170 | 0.314 | | 0.435 | | 124.506 | 0.875 | 0.094 | 0.550 |
| 30-60 | 0.210 | 0.349 | | 0.449 | | 66.585 | 0.450 | 0.034 | 0.734 |
| 60-90 | 0.237 | 0.367 | | 0.458 | | 49.598 | 0.267 | 0.024 | 0.754 |
| 90-120 | 0.223 | 0.353 | | 0.443 | | 46.631 | 0.200 | 0.020 | 0.784 |
| 120-150 | 0.210 | 0.340 | | 0.430 | | 43.823 | 0.100 | 0.016 | 0.826 |
| 150-180 | 0.200 | 0.320 | | 0.420 | | 13.103 | 0.070 | 0.014 | 0.999 |
| 180-210 | 0.197 | 0.317 | | 0.417 | | 10.800 | 0.050 | 0.013 | 0.999 |
| 210-250 | 0.190 | 0.304 | | 0.410 | | 8.711 | 0.010 | 0.011 | 0.999 |

| Depth | LL15 | DUL | SAT | KS | OC | Fbiom | Finert |
| --- | --- | --- | --- | --- | --- | --- | --- |
| cm | -----------mm/mm------------- | | | mm/d | % | 0-1 | 0-1 |
|  | **Calibration Experiment 5 (Rhine et al., 2010)** | | | | | | |
| 0-30 | 0.307 | 0.434 | 0.515 | 31.433 | 1.170 | 0.093 | 0.466 |
| 30-60 | 0.377 | 0.483 | 0.567 | 31.300 | 0.600 | 0.035 | 0.814 |
| 60-90 | 0.419 | 0.510 | 0.609 | 49.733 | 0.163 | 0.024 | 0.862 |
| 90-120 | 0.396 | 0.495 | 0.594 | 52.133 | 0.143 | 0.020 | 0.902 |
| 120-150 | 0.364 | 0.476 | 0.575 | 52.100 | 0.080 | 0.016 | 0.949 |
| 150-180 | 0.348 | 0.467 | 0.564 | 50.800 | 0.020 | 0.014 | 0.999 |
| 180-210 | 0.348 | 0.467 | 0.564 | 50.733 | 0.013 | 0.013 | 0.999 |
| 210-250 | 0.348 | 0.467 | 0.564 | 50.600 | 0.000 | 0.012 | 0.999 |
|  | **Testing Experiment 1 (Archontoulis et al., 2019)** | | | | | | |
| 0-30 | 0.153 | 0.259 | 0.407 | 146.000 | 2.260 | 0.089 | 0.476 |
| 30-60 | 0.106 | 0.215 | 0.335 | 139.500 | 1.460 | 0.035 | 0.814 |
| 60-90 | 0.130 | 0.263 | 0.393 | 133.000 | 0.730 | 0.025 | 0.860 |
| 90-120 | 0.128 | 0.227 | 0.393 | 388.000 | 0.490 | 0.019 | 0.999 |
| 120-150 | 0.090 | 0.200 | 0.393 | 388.000 | 0.490 | 0.016 | 0.999 |
| 150-180 | 0.090 | 0.200 | 0.285 | 54.236 | 0.490 | 0.014 | 0.999 |
| 180-210 | 0.090 | 0.200 | 0.273 | 33.110 | 0.490 | 0.013 | 0.999 |
| 210-250 | 0.090 | 0.200 | 18.638 | 0.412 | 0.012 | 0.999 | 0.999 |
|  | **Testing Experiment 2 (Oosterhuis et al., 1990)** | | | | | | |
| 0-30 | 0.115 | 0.297 | 0.434 | 152.000 | 0.875 | 0.060 | 0.406 |
| 30-60 | 0.126 | 0.340 | 0.456 | 104.000 | 0.455 | 0.025 | 0.763 |
| 60-90 | 0.203 | 0.377 | 0.494 | 88.667 | 0.440 | 0.020 | 0.849 |
| 90-120 | 0.257 | 0.388 | 0.478 | 58.000 | 0.440 | 0.020 | 0.852 |
| 120-150 | 0.255 | 0.385 | 0.473 | 58.000 | 0.270 | 0.020 | 0.885 |
| 150-180 | 0.250 | 0.384 | 0.472 | 50.000 | 0.090 | 0.020 | 0.999 |
| 180-210 | 0.250 | 0.383 | 0.469 | 49.000 | 0.033 | 0.020 | 0.999 |
| 210-250 | 0.250 | 0.381 | 0.466 | 47.000 | 0.014 | 0.020 | 0.999 |

Supplementary Table 3 Cultivar Parameters for each experiment.

| Cultivar Parameters | Units | | Array of Values |
| --- | --- | --- | --- |
| **Calibration Experiments 1 and 2 (Scott et al., 1989)-Maturity Group 4** | | | |
| node_sen_rate | | °C-days/node | 160 |
| x_pp | | Daylight | 13.09, 13.9, 14.8, 15.6 |
| tt_end_of_juvenile | | °C-days | 150, 175, 200, 400 |
| tt_floral_initiation | | °C-days | 320, 350, 380, 760 |
| tt_flowering | | °C-days | 390, 420, 527, 1404 |
| tt_start_grain_fill | | °C-days | 535, 713, 1069, 2852 |
| **Calibration Experiment 3 (Scott et al., 1990)-Maturity Group 4** | | | |
| node_sen_rate | | °C-days/node | 160 |
| x_pp | | Daylight | 13.09, 13.9, 14.8, 15.6 |
| tt_end_of_juvenile | | °C-days | 100, 133, 200, 400 |
| tt_floral_initiation | | °C-days | 130,253, 380, 760 |
| tt_flowering | | °C-days | 263,351, 527, 1404 |
| tt_start_grain_fill | | °C-days | 610, 713, 1069, 2852 |
| **Calibration Experiment 4 (Board et al., 2008)-Maturity Group 5** | | | |
| node_sen_rate | | °C-days/node | 160 |
| x_pp | | Daylight | 12.83, 13.7, 14.5, 15.3 |
| tt_end_of_juvenile | | °C-days | 100, 133, 200, 400 |
| tt_floral_initiation | | °C-days | 180, 307, 460, 920 |
| tt_flowering | | °C-days | 271, 361, 541, 1443 |
| tt_start_grain_fill | | °C-days | 620, 732, 1099, 2929 |
| **Calibration Experiment 5 (Rhine et al., 2010)-Maturity Group 4** | | | |
| node_sen_rate | | °C-days/node | 160 |
| x_pp | | Daylight | 13.09, 13.9, 14.8, 15.6 |
| tt_end_of_juvenile | | °C-days | 75, 100, 200, 400 |
| tt_floral_initiation | | °C-days | 140, 200, 380, 760 |
| tt_flowering | | °C-days | 263, 351, 527, 1404 |
| tt_start_grain_fill | | °C-days | 615, 750, 1069, 2852 |
| **Testing Experiment 1 (Archontoulis et al., 2019)-Maturity Group 3** | | | |
| node_sen_rate | | °C-days/node | 120 |
| x_pp | | Daylight | 13.59, 14.6, 15.6, 16.6 |
| tt_end_of_juvenile | | °C-days | 100, 133, 200, 400 |
| tt_floral_initiation | | °C-days | 128, 171, 256, 512 |
| tt_flowering | | °C-days | 266, 348, 492, 1312 |
| tt_start_grain_fill | | °C-days | 510, 676, 999, 2664 |
| Cultivar Parameters | | Units | Array of Values |
| **Testing Experiment 2 (Oosterhuis et al. 1990)-Maturity Group 5** | | | |
| node_sen_rate | | °C-days/node | 130 |
| x_pp | | Daylight | 12.83, 13.7, 14.5, 15.3 |
| tt_end_of_juvenile | | °C-days | 110, 143, 210, 410 |
| tt_floral_initiation | | °C-days | 230, 307, 460, 920 |
| tt_flowering | | °C-days | 271, 361, 541, 1443 |
| tt_start_grain_fill | | °C-days | 463, 695, 950, 2850 |
| **Testing Experiment 3 (Oosterhuis et al. 1990)-Maturity Group 5.5** | | | |
| node_sen_rate | | °C-days/node | 130 |
| x_pp | | Daylight | 12.83, 13.7, 14.5, 15.3 |
| tt_end_of_juvenile | | °C-days | 110, 143, 210, 410 |
| tt_floral_initiation | | °C-days | 230, 307, 460, 920 |
| tt_flowering | | °C-days | 271, 361, 541, 1443 |
| tt_start_grain_fill | | °C-days | 600, 778, 1166, 3110 |
